# Novel mutations c.28G>T (p.Ala10Ser) and c.189G>T (p.Glu63Asp) in WDR62 associated with early onset acanthosis and hyperkeratosis in a patient with autosomal recessive microcephaly type 2

**DOI:** 10.18632/oncotarget.13279

**Published:** 2016-11-10

**Authors:** Santasree Banerjee, Huishuang Chen, Hui Huang, Jing Wu, Zhiyun Yang, Weiping Deng, Dongna Chen, Jianlian Deng, Yan Su, Yang Li, Chao Wu, Ye Wang, Hao Zeng, Yiming Wang, Xunhua Li

**Affiliations:** ^1^ BGI-Shenzhen, Shenzhen, China; ^2^ Department of Medical Imaging, 1st affiliated hospital, Sun Yat-sen University, Guangzhou, PR China; ^3^ Department of Dermatology, Guangdong General Hospital, Guangdong Academy of Medical Sciences, PR China; ^4^ Department of Medical Genetics, Center for Genome Research, Sun Yat-sen University, Guangzhou, PR China; ^5^ Xinhua College, Sun Yat-sen university, Guangzhou, PR China; ^6^ Department of Neurology, 1st affiliated hospital, Sun Yat-sen University, Guangzhou, PR China

**Keywords:** microcephaly 2, WDR62 mutation, compound heterozygosity, novel mutations, exome sequencing, Pathology Section

## Abstract

Microcephaly (MCPH) is a developmental disorder characterized by reduced brain size and intellectual disability. A proportion of microcephaly is caused by defects in a single gene. Microcephaly 2 (MCPH2) is one of the most frequent subtypes of MCPH.WD repeat-containing protein 62 gene (*WDR62*) is the most frequently mutated gene in MCPH2 patients. Phenotypes involving dermatological changes in MCPH2 have not been reported. We have identified and investigated a 5-year-old Chinese girl with markedly reduced brain size (86% of normal size), intellectual disability and psychomotor developmental delay. The patient also exhibited spattered blisters and reduced hair density on her head, anisochromasia with reticular hyperpigmentation and hypopigmentation on the trunk, which she has had since the age of 4 and had been found by her parents. Histological examination of a skin biopsy revealed acanthosis, hyperkeratosis and necrotic keratinocytes. Whole exome and Sanger sequencing identified two novel missense mutations, c.28G>T and c.189G>T, in the *WDR62* gene. Both the mutations non-synonymously affect evolutionarily conserved amino acids and are predicted to be disease causing. We report the first case of MCPH2 that also presented with marked dermatological changes. Our findings expand the mutational and phenotypical spectra of MCPH2 and are valuable in the mutation-based pre- and post-natal screening and genetic diagnosis for MCPH2.

## INTRODUCTION

Microcephaly (MCPH) is a disorder of fetal brain growth; individuals with microcephaly have small brains and almost always have intellectual disability. The etiology of microcephaly can be hereditary or environmental, as with the Zika virus-related MCPH. Hereditary microcephaly constitutes a large proportion of the cases, with 17 loci registered in the Online Mendelian Inheritance in Man database (MIM, omim.org/), and each is caused by a distinct gene. However, most subtypes of MCPH have overlapping phenotypes. Dermatological involvement in MCPH has only been reported in two patients, who had prenatal and postnatal growth retardation, microcephaly, developmental delay, generalized reticular hyperpigmentation, hypohidrosis, with absence of fingertip prints and palmoplantar hyperkeratosis [1]. However, the etiology of these two cases was unidentified.

Autosomal recessive primary microcephaly 2 (MCPH2) [MIM#604317] has a prevalence between 1 in 30,000 and 1 in 250,000 [2]. It is the second most common subtypes of MCPH [3]. The clinical diagnosis of MCPH2 is based on a head circumference more than 3 standard deviations (SD) below the age- and sex-matched population mean and mental retardation with exclusion of other causes [4]. The WD repeat-containing protein 62 (*WDR62*) gene has been identified as a genetic cause of MCPH2. Patients with *WDR62* mutations present with a wide spectrum of cortical malformations including cortical thickening, polymicrogyria, abnormal/simplified gyral pattern, pachygyria, hypoplasia of the corpus callosum, and schizencephaly, heterotopias [5]. Some patients also have evidence of lissencephaly, cerebellar hypoplasia, and hippocampal dysmorphy [6, 7, 8]. A broad range of neurological and behavioral manifestations have been reported, including decreased fetal movements, delayed psychomotor development, mental retardation, impulsivity and aggression [9]. Phenotypes involving dermatological symptoms have not been reported in MCPH2. In this paper, we report our investigation of a Chinese girl who exhibited marked dermatological phenotypes and the associated two novel compound heterozygous mutations in *WDR62*.

## RESULTS

### Clinical findings

**Figure 1:**
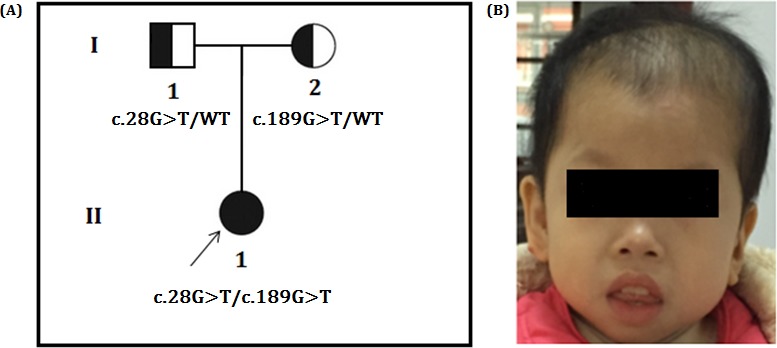
**A.** Pedigree of the family. The filled symbol indicates the patient (proband), and the half-filled symbols show the carrier parents, who were heterozygous carriers but were unaffected. The arrow points to the proband. **B.** Facial phenotype of the patient, which shows the sloping forehead, the convex facial profile, the full lips, and the small chin. The appearance of low-set and posteriorly rotated ears in the lateral picture is partly due to reclamation of the head. It also shows the reduced hair on the top of the head.

Proband is a 5-year-old girl, the first child of non-consanguineous healthy parents of Han Chinese (Figure 1A), presented with psychomotor retardation since infancy. She was unable to raise her head and sit without external support until she was 3 years old. She could not walk independently. She could not say any words except “mom” and “dad.” Feeding had been difficult due to frequent vomiting, constipation, anemia and recurrent infection of the respiratory tract. Hair was easily lost, resulting in scant hair on the top of the head. However, seizures had not been observed.

**Figure 2:**
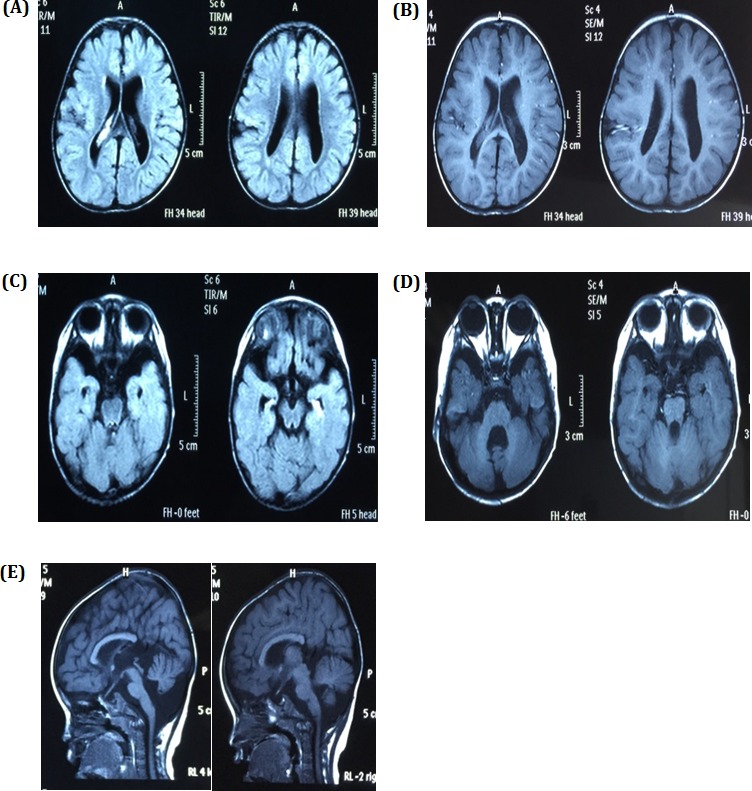
Brain images **A.** and **B.** showing the expansion of bilateral brain ventricles, dysplasia of the brain white matter and suspicious schizencephaly in the right parietal lobe, **C.** dysplasia of the temporal lobe with small hippocampus and enlarge temporal horn, **D.** obvious expansion of the fourth ventricle and slight atrophy of cerebellum, and **E.** slight atrophy of the brain stem and thinning of the corpus callosum with absence of the splenium.

Our physical examinations showed that she was conscious but apathetic. She could not communicate verbally. Her height was 73 cm, and her weight was 6 kg. Her head circumference was only 43 cm, which is 86% of the normal range. She was hypomyotonic and had scoliosis. She had facial features typical of MCPH2 (Figure 1B), which manifest in a wide and low bridge of the nose, thick lips, broad eye distance, sloping forehead, high palate-maxillary arch and low-set and large ears. Brain imaging showed a slight expansion of bilateral brain ventricles, which was apparent on the left side; obvious expansion of the fourth ventricle; thinning of the corpus callosum with the absence of the splenium; dysplasia of the temporal lobe with small hippocampus, enlarged temporal horn and broadening lateral fissure; leukodystrophy, dysplasia of the white matter; and suspected schizencephaly in the right parietal lobe and slight atrophy of the brain stem and cerebellum. (Figure 2A-2E). Abdominal ultrasound examinations showed that her liver measured 1.6 cm under the ribs. Her kidneys were 4 × 2.2 cm on the right side and 3.8 × 2.3 cm on the left side.

**Figure 3:**
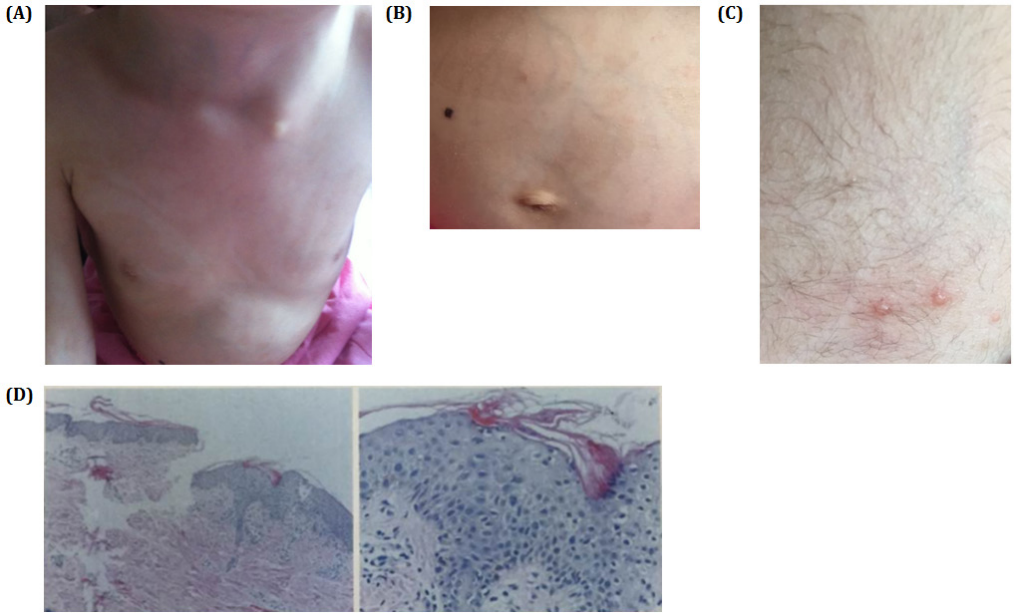
**A.** and **B.** Pictures show spattered pigmentation on the trunk. **C.** Three blisters on the skin. **D.** HE slides of the skin biopsy showing hyperkeratosis in the skin and porokeratosis. Stratum spinosum thickened slightly with edema in intracellular and intercellular areas. Pigment granules in the basal layer with increased punctate liquefaction degeneration. Few lymphocytes infiltrate beside blood vessel in the shallow corium layer.

What particularly caught our attention were the unusual skin lesions, the spattered blisters and the reduced hair density (Figure 3A) on her head and the anisochromasia with reticular hyperpigmentation and hypopigmentation on the trunk (Figure 3B, 3C) that had first been observed by her parents when she was four years old. Her nails and mucous membranes appeared normal. Histological examination of skin biopsy revealed acanthosis, hyperkeratosis and necrotic keratinocytes. There was melanin in melanophages in the upper dermis (Figure 3D, 3E). Based on the results of our clinical and genetic testing, we diagnosed her with MCPH2.

Written informed consent was obtained from the parents. The project was approved by the ethics committee of the BGI and in accordance with the Principles of the Declaration of Helsinki.

### Karyotyping and chromosomal array analysis excluded chromosomal abnormality and pathogenic copy number variation

Karyotyping of the patient indicated the presence of a normal set of chromosomes. Chromosomal array analysis showed a 476 kb duplication at 14q32.33 (106270453-106746712). This duplication has been classified as a benign polymorphism by the Database of genomic variation (DGV, dgv.tcag.ca) and Phenotype in Humans using Ensembl Resources (DECIPHER, decipher.sanger.ac.uk).

### Whole exome sequencing and Sanger sequencing identified two novel mutations in WDR62

**Figure 4:**
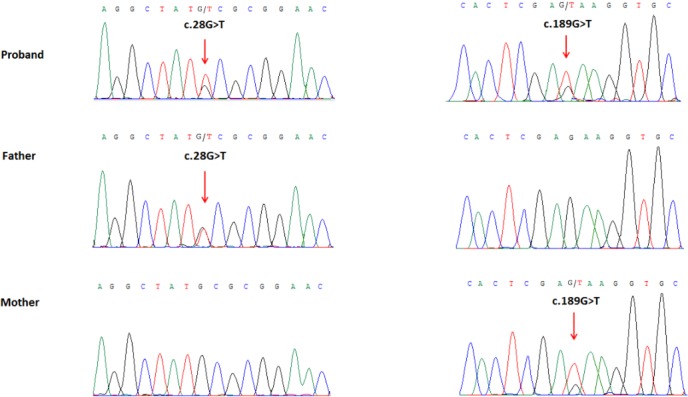
Partial DNA sequences in the WDR62 by Sanger sequencing of the family Upper line: the proband, middle line: the father, bottom line: the mother. Arrows point to the mutations. The proband inherited both c.28G>T and c.189G>T mutations. The father carries the c.28G>T mutation, and the mother carries the c.189G>T mutation.

We performed whole exome sequencing of DNA from the proband and her parents. Whole exome sequencing identified two novel heterozygous mutations of the *WDR62* gene in the proband: a missense mutation (c.28G>T) in exon 1 that was inherited from the father, which replaces alanine with serine (p.Ala10Ser) in amino acid 10, and another missense mutation (c.189G>T) in exon 2, inherited from the mother, which results in substitution of glutamic acid by aspartic acid (p.Glu63Asp) in amino acid 63. We did not find deleterious mutations in any other genes that underlie MCPH. The two mutations were further confirmed by Sanger sequencing (Figure 4). The two mutations were absent in the Human Gene Mutation database (HGMD, www.hgmd.cf.ac.uk/) and MIM. The mutation c.28G>T was not found in BGI's database, which has ~ 30,000 Chinese Han samples and the frequency of the other mutation, c.189G>T was 0.027, all the mutations were heterozygous.

### Across-species conservation analysis showed that the two mutations occurred in conserved residues

**Figure 5:**
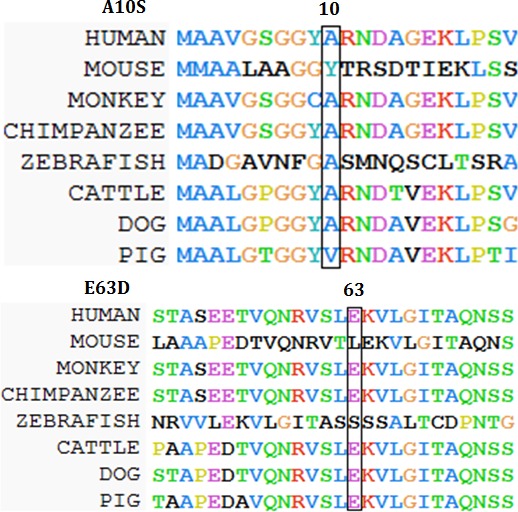
Amino acid alignment of the wild-type protein encoded by *WDR62* (*Homo sapiens*) (GenBank Accession: NM_001083961.1) with mouse (*Mus musculus*) (GenBank Accession: NM_146186.3), rhesus monkey (*Macaca mulatta*) (GenBank Accession: AFH29290.1), chimpanzee (*Pan troglodytes*) (GenBank Accession: JAA38944.1), zebrafish (*Danio rerio*) (GenBank Accession: CM002899.1), cattle (*Bos taurus*) (GenBank Accession: GK000018.2), dog (*Canis lupus familiaris*) (GenBank Accession:CM000001.3), and pig *(Sus scrofa*) (GenBank Accession: CM000817.4). The two boxes show that amino acids 10 and 63 are conserved across these species.

Multiple sequence alignment showed that both p.Ala10 and p.Glu63 in *WDR62* are evolutionarily highly conserved among different species, indicating their importance in the functions of the wild-type *WDR62* protein (Figure 5).

### *In silico* analysis indicated the pathogenic nature of the mutations

Mutation Taster [10] predicted these two novel mutations to be “disease causing”. PolyPhen-2 [11] predicted them to be “probably damaging”, and I-MUTANT 2.0 [12] predicted that these mutations would result in “decreasing the stability of the *WDR62* protein”.

## DISCUSSION

We identified two novel missense mutations in *WDR62* gene: a single nucleotide transversion c.28G>T in exon 1, inherited from the unaffected father, and a single nucleotide transition c.189G>T in exon 2, inherited from the healthy mother. Mutation Taster predicted these two novel variants to be “disease causing”. These two mutations are also predicted to be “decreasing the stability of the *WDR62* protein” by I-MUTANT 2.0. Both the mutations (p.10Ala and p.63Glu) affect evolutionarily conserved amino acids. In addition, the replacement of nonpolar/neutral alanine by polar/neutral serine may well affect the protein structure encoded by the wild-type *WDR62.* According to the guidelines of American College of Medical Genetics and Genomics (ACMG) [13], these two mutations could be classified as “likely pathogenic”.

Our array-CGH analysis showed that the patient carried a 476 kb duplication of 14q32.33 (106270453-106746712), which contains the AK128652, KIAA0125, ADAM6 and LINC00226 genes. There has been no report showing that these genes have known functions in brain development or associated with dermatological phenotypes. duplication has been classified as a “benign polymorphism” by the most widely used CNV databases (DECIPHER and DGV) in clinical genetics practice. We tried to persuade the patient's parents to undergo this analysis. Unfortunately, they rejected consent for another blood sample to be taken for this analysis; therefore the origin of this duplication in the patient is currently unknown.

*WDR62* is a scaffold protein of the c-Jun N-terminal kinase (JNK) pathway; it encompasses a WD40 domain, an MKK7 binding domain, and a JNK docking domain. *WDR62* co-localizes to the mitotic spindle poles during early stages of the cell cycle. *WDR62* regulates spindle orientation, centrosome integrity, and progression through mitosis [14]. *WDR62* is essential for mitotic spindle stabilization during mitosis, and it accumulates at the centrosome or the nucleus in a cell-cycle-dependent manner [14]. Mutant *WDR62* proteins fail to localize to the mitotic spindle pole [15]. Presently, 31 mutations of the *WDR62* gene have been reported in MCPH2. However, apart from the MCPH2-associated symptoms, our patient also showed hyperkeratosis with typical abnormal skin pigmentation and blisters. Furthermore, in our whole exome sequencing data, we did not identify any deleterious mutations that could cause the reported dermatological phenotypes. Our findings in the skin seem to echo two previously reported MCPH cases, although not MCPH2 cases, which also presented with dermatological lesions [1]. One possible explanation for the dermatological phenotypes in our patient is that the two novel mutations may cause the loss of the afore mentioned physiological functions of the wild type protein, thereby inducing the dermatological changes. This suggests that *WDR62* may play a role in the normal development and maintenance of skin. However, this explanation needs to be further confirmed by other cases and the exact mechanisms need further investigations.

In conclusion, we report the first case of MCPH2 in a patient who presented with early onset acanthosis and hyperkeratosis, with two underlying novel mutations in the *WDR62* gene. Our study not only expands the mutational spectrum for *WDR62*-associated MCPH2 but is also valuable for the mutation-based pre- and post-natal screening and genetic diagnosis of MCPH2.

## MATERIALS AND METHODS

### Karyogram and chromosomal array analyses

Standard G-banding karyotyping was performed. Chromosome microarray analysis was performed using a CytoScan HD array (Affymetrix). The procedures for DNA digestion, ligation, PCR amplification, fragmentation, labeling and hybridization to the array were performed according to the manufacturer's protocols (Affymetrix). The data were analyzed using Chromosome Analysis Suite software version 1.2.2, and the reporting threshold of the copy number was set at 10 kb, with marker count at ≥50, as we previously reported [16].

### Whole exome sequencing and mutation selection

Genomic DNA was extracted from peripheral blood using a QIAamp DNA Blood Mini Kit (Qiagen, Hilden, Germany) according to the manufacturer's instructions. All three family members (parents, proband) were subjected to exome sequencing. Sequences were captured by Agilent SureSelect version 4 (Agilent Technologies, Santa Clara, CA) according to the manufacturer's protocols. The enriched library was sequenced on an Illumina HighSeq2000. The sequencing reads were aligned to GRCh37.p10 using Burrows-Wheeler Aligner software (version 0.59). We then performed local realignment and base quality recalibration of the Burrows-Wheeler aligned reads using the GATK IndelRealigner and the GATK BaseRecalibrator, respectively (broadinstitute.org/). Single-nucleotide variants (SNV) and small insertions or deletions (indel) were identified by the GATK UnifiedGenotyper (broadinstitute.org/). Variants were annotated using the Consensus Coding Sequences Database (20130630) at the National Center for Biotechnology Information.

**Figure 6:**
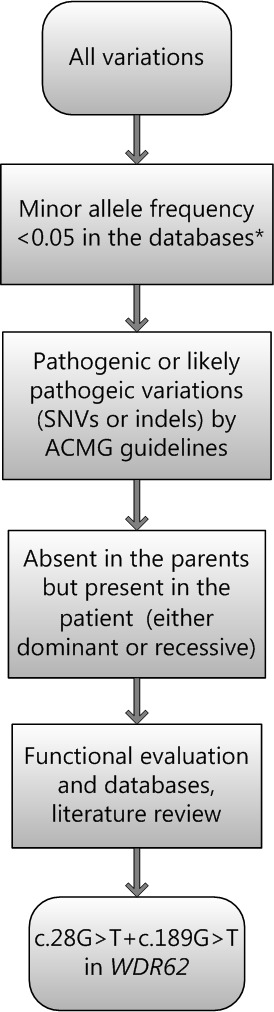
Filtering process for pathogenic mutations in all variations obtained by exome sequencing *Databases used: dbSNP, Hapmap, 1000 Genomes Project and BGI's in-house database of ~30000 Chinese people. SNV: single nucleotide variation. Indel: small insertion and deletion.

We selected variations obtained from exome sequencing with minor allele frequencies <0.05 in any of the following databases (dbSNP, Hapmap, 1000 Genomes Project) and our in-house database for ~30,000 Chinese Han samples. We also selected pathogenic and likely pathogenic variations according to the ACMG guidelines [13]. We further compared the remaining deleterious variations in the patient with variations carried by her unaffected parents and the gene's functions with the references of OMIM and literature. For the detailed filtering process, please see Figure 6.

### Sanger sequencing

To validate putative mutations, Sanger sequencing was performed. Primers flanking the candidate loci were designed based on the reference genomic sequences of the Human Genome from GenBank in NCBI and synthesized by Invitrogen, Shanghai, China. PCR amplification was carried out in an ABI 9700 Thermal Cycler. PCR products were directly sequenced on an ABI PRISM 3730 automated sequencer (Applied Biosystems, Foster City, CA, USA). Sequence data comparisons and analysis were performed by DNASTAR SeqMan (DNASTAR, Madison, Wisconsin, USA).

The compound heterozygous mutations identified through whole exome sequencing were verified through Sanger sequencing using the following primers: F1 5′-ACCCCATGACGCTTAATCAGGC-3′, R1 5′-GCAGCTTCTCACCCGGTTCTG-3′, F2 5′-AGAAGCTCAGTGTGGGTGTTGAAT-3′, and R2 5′-ACAGAGAGGGGGCTATGGAATC-3′. The reference sequence NM_001083961.1 of WDR62 was used.

### Bioinformatics analysis

#### Evolutionary conservation test

To understand the evolutionary conservation of the wild type amino acids of the two novel mutations of *WDR62* gene, we used ClustalW2 [17] to perform sequence alignment between human, mouse (*Mus musculus*) (GenBank Accession: NM_146186.3), rhesus monkey (*Macaca mulatta*) (GenBank Accession: AFH29290.1), chimpanzee (*Pan troglodytes*) (GenBank Accession: JAA38944.1), zebrafish (*Danio rerio*) (GenBank Accession: CM002899.1), cattle (*Bos taurus*) (GenBank Accession: GK000018.2), dog (*Canis lupus familiaris*) (GenBank Accession:CM000001.3), and pig (*Sus scrofa*) (GenBank Accession: CM000817.4).The two boxes show that amino acids 10 and 63 are conserved across these species (Figure 5).

#### *In silico* prediction

To analyze the effects of the missense mutations on the functions of the WDR62 protein, we used several bioinformatics programs, viz. MutationTaster [10], PolyPhen2 [11] and I-MUTANT 2.0 [12]. These bioinformatics programs predict the effect of missense mutations on the basis of overall stability, pathogenicity and evolutionary conservation. We used these programs as fundamental and preliminary analyses designed to investigate the molecular mechanism behind the disease phenotype.
